# Localized Bone Loss Resulted from an Unlikely Cause in an 11-Year-Old Child

**DOI:** 10.1155/2018/3484513

**Published:** 2018-07-08

**Authors:** Bianca Tozi Portaluppe Bergantin, Daniela Rios, Daniela Silva Barroso Oliveira, Edmêr Silvestre Pereira Júnior, João Adolfo Costa Hanemann, Heitor Marques Honório

**Affiliations:** ^1^Department of Pediatric Dentistry, Orthodontics and Public Health, Bauru School of Dentistry, University of São Paulo, Bauru, SP, Brazil; ^2^Department of Clinic and Surgery, Federal University of Alfenas, Rua Gabriel Monteiro da Silva, 700, 37130000 Alfenas, MG, Brazil; ^3^Department of Clinic and Surgery, Pediatric Dentistry, Federal University of Alfenas, Rua Gabriel Monteiro da Silva, 700, 37130000 Alfenas, MG, Brazil

## Abstract

Periodontal diseases have several causes, amongst them, by foreign bodies. In this case report, an 11-year-old child who lived in a rural area and has never been treated by a dentist presented an extensive horizontal bone loss and edema on the region of tooth 44. The diagnosis of foreign body was obtained after biopsy, since an elastic band around the middle of the root tooth was found. The elastic band was not radiopaque, and the patient did not inform that she found the elastic band on the floor of the school and introduced the tooth by herself. Based on the case reported, it is concluded that anamnesis and clinical and radiographic examination are fundamental strategies to obtain the diagnosis, but sometimes, especially in children, there may be inconsistencies that can be elucidated by a biopsy.

## 1. Introduction

The term “periodontal disease” includes every pathological condition that causes damages to support and/or protection periodontium [1, 2]. Basically, these damages start with an inflammation of these tooth-supporting tissues [3], followed by the differentiation of osteoclasts induced by inflammatory cells that degrade the mineralized matrix, generating bone resorption [4], causing exposure of the roots, mobility, and tooth loss [5].

Periodontal diseases are not limited to adults [5, 6], and the occurrence of radiographic bone loss in children (2–11 years) has a significant prevalence (8.88%) [7]. The inflammation that generates bone resorption may be the consequence of several factors, among them the foreign body reaction that is a local gingival inflammatory condition due to the introduction of foreign materials into the gingival connective tissue, causing ruptures in the epithelium. The most common examples of foreign bodies in the literature are amalgam restorations or penetration of materials during clinical procedures [8], such as elastic bands for tooth separations for orthodontic treatment [9].

The literature describes bone loss and mobility resulted from orthodontic elastic bands along the roots [10–17]. The early diagnosis improves the chance for a successful treatment [18]; however, when the patient reports no previous orthodontic treatment and the elastic band is radiolucent, it is very difficult to diagnose. This case report is of interest in that an 11-year-old child, who lived in a rural area, has never been treated by a dentist, presented an extensive horizontal bone loss and vestibular and lingual edema on the region of tooth 44, caused by an elastic band.

## 2. Case Report

**Figure 1:**
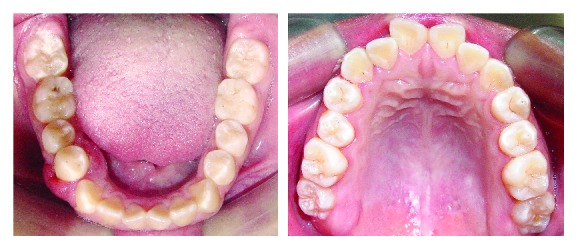
Adequate oral hygiene aspect.

**Figure 2:**
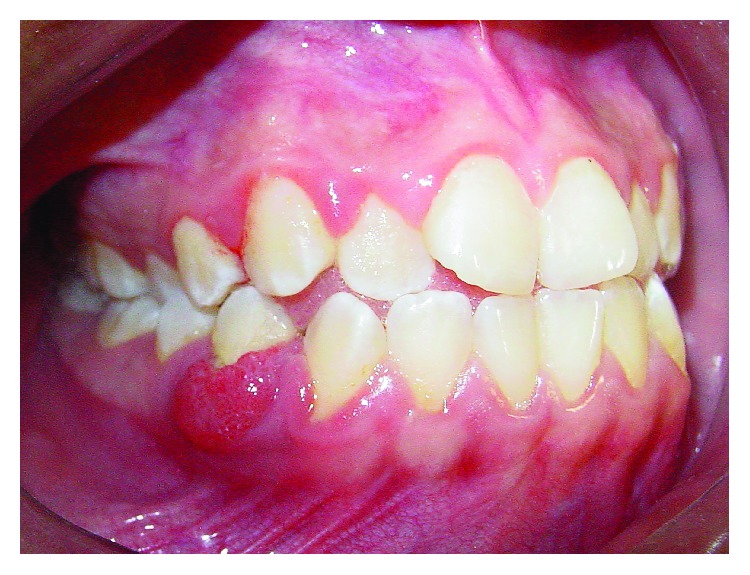
Edema around the 44 region.

**Figure 3:**
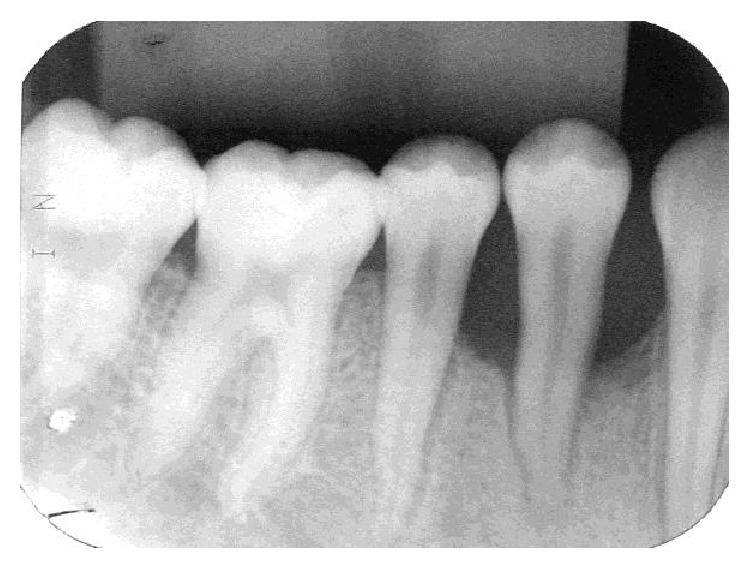
The radiographic image, where we can observe the extensive horizontal bone loss.

**Figure 4:**
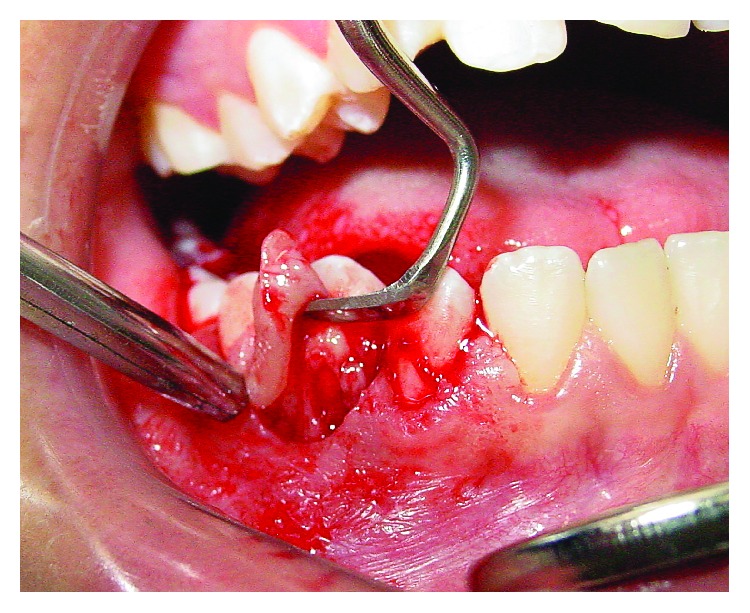
Excisional biopsy.

**Figure 5:**
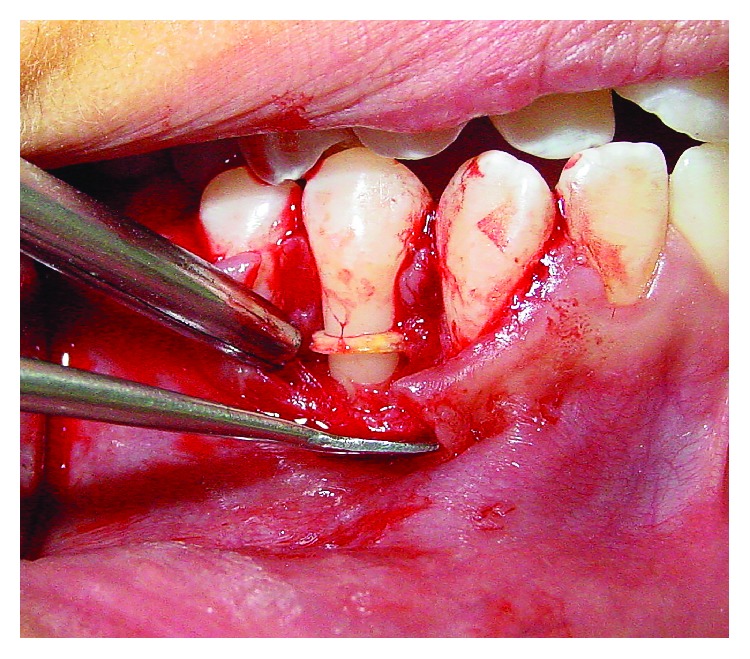
The orthodontic elastic band found.

**Figure 6:**
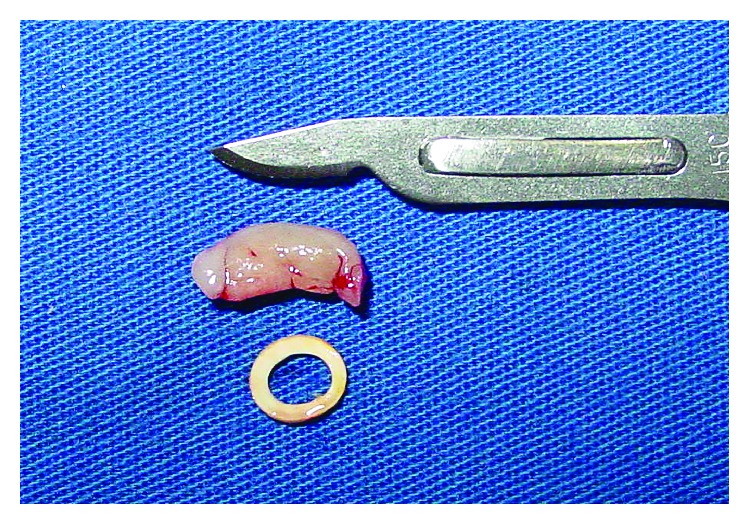
The elastic band and the tissue removed.

**Figure 7:**
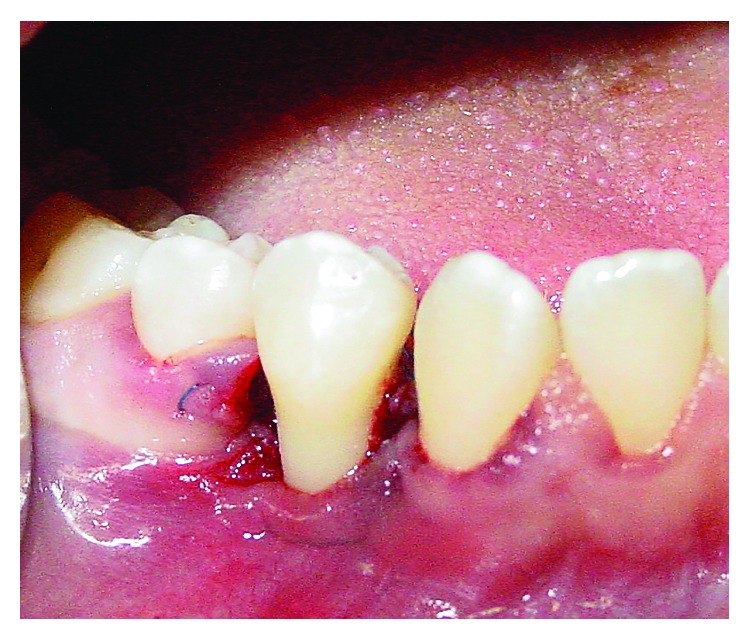
Immediate postoperative aspect.

**Figure 8:**
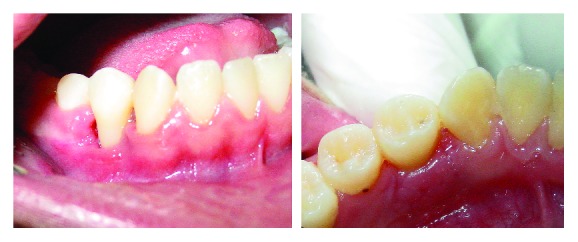
Aspect after seven days postoperative.

An 11-year-old girl with no pain complaint and adequate oral hygiene reported mobility on the 44 teeth (Figure 1). Clinically, a reddish edema around the teeth, sessile, with an irregular surface, and no local irritant was found (Figure 2). In the anamnesis, the parents reported that the child had never been to a dentist. In addition, the child said that she did not put any object in the affected region. The radiography showed an extensive horizontal bone loss on the mesial and distal areas of tooth 44 (Figure 3). After clinical examination and anamnesis, the probable diagnosis of *pyogenic granuloma* was discarded because no trauma or local irritant [19] was found or reported. In the first visit, the professional irrigated the site with sodium iodide 2% and hydrogen peroxide, and beyond that, subgingival scaling was made. After these procedures, no foreign body was removed or identified. Therefore, a biopsy and the granuloma removal were planned in the next visit. The surgery started with anesthesia of the alveolar, lingual, and buccal nerve block, incision with scalpel blade, and tissue removal by excisional biopsy (Figure 4). During the surgery, the foreign body, an orthodontic elastic band, was found around the root's tooth (Figure 5). The elastic band was removed (Figure 6), the root scaling was performed, and the soft tissues were sutured (Figure 7). After 7 days, the patient returned for the suture removal, showing adequate healing (Figure 8). The patient never attended to the subsequent control schedules.

## 3. Discussion

Bone loss can be caused by accumulation of bacteria that release lipopolysaccharides that are recognized by toll-like receptors, initiating a signaling cascade and inducing secretion of proinflammatory proteins. However, this causal factor is rarer in children, being the more common bone loss caused by foreign body reaction, in which the presence of foreign materials lead to rupture of the epithelium and contact with the connective tissue generating local inflammatory reactions. In any of these cases, bone loss is a consequence of a degradation of tissue matrix proteins and immunoglobulins due to the differentiation of osteoclasts precursors in osteoclasts, causing a bone resorption and apical migration of the tissue (consequently of the foreign body) [2, 4]. According to the American Academy of Pediatric Dentistry, bone loss can also occur in the absence of local factors such as aggressive periodontitis, which is considered a disease of adolescents and young adults, and it can begin at any age and usually affects the entire dentition, with a generally genetic cause. In the case presented, the patient presented gingival edema witch discarded the diagnosis of pyogenic granuloma [20] by the presence of a periodontal pocket, with horizontal bone loss observed radiographically. However, clinically, it was not possible to observe the presence of supra and subgingival biofilm, which excluded the hypothesis of aggression by the presence of bacteria; on the other hand, as the bone loss was located in a single dental element, the hypothesis of the presence of periodontal disease was also eliminated. Therefore, the remaining possible causal factor was foreign body or other type of oral lesion.

Orthodontic elastics are widely used for diastema correction, separation of teeth for band placement, and crossbite correction [21, 22]. These are left in place for a maximum of 1 week [19]. In cases where the elastic band is left over for a longer period, iatrogenic situations may occur [9], in which there is a great loss of bone and, in more severe cases, leads to loss of the dental element [19]. In the clinical case presented, the patient was from a rural area and reported having no previous experience with dental care, which made the hypothesis on the iatrogenic placement of the elastic discarded, since in the cases reported in the literature this was due to failure of the surgeon dentist [9–14]. Given the difficulty of diagnosis through clinical and radiographic examination, it was decided to perform the biopsy for histological analysis of the specimen. However, during the surgery, the presence of an orthodontic elastic band was detected around the tooth. After the dentist questioned the child of whom would have put the elastic band, she reported that she had forgotten to say it in the anamnesis, but she herself had put the elastic band found on the school floor.

In view of the above, we can observe that even if a good anamnesis is performed, children are often not able to adequately report the facts, due to oblivion or omission. Another point to be discussed is the quality of the orthodontic elastic band, which ideally must be radiopaque, allowing its detection in the radiographic exam. In addition, it might be also nontoxic, reducing the tissue adverse reaction [23]. However, this type of material is often illegally marketed, not following quality standards. In addition, the elastics are being used widely, due to the higher prevalence of malocclusions and, consequently, higher necessity of orthodontic treatment [24], causing unlikely use, as in the present case in which the child placed the elastic band, found on the school floor, which is equal to children who used appliance.

Although the biopsy, especially in children, seems to be a very invasive treatment, there are situations in which it is indicated and fundamental for the diagnosis. However, oftentimes, professionals opt for less invasive treatments, delaying the procedure, which can lead to worsening of the condition, making it difficult to treat [22]. Based on the case reported, it is concluded that anamnesis and clinical and radiographic examination are fundamental strategies to obtain the diagnosis, but sometimes, especially in children, there may be inconsistencies that can be elucidated by a biopsy.
